# The peripheral origin of tap-induced muscle contraction revealed by multi-electrode surface electromyography in human vastus medialis

**DOI:** 10.1038/s41598-020-59122-z

**Published:** 2020-02-10

**Authors:** Alberto Botter, Taian M. Vieira, Tommaso Geri, Silvestro Roatta

**Affiliations:** 1 Laboratory for Engineering of the Neuromuscular System (LISiN), Department of Electronics and Telecommunications, Politecnico di Torino, Corso Duca degli Abruzzi 24, 10129 Turin, Italy; 2 PoliToBIOMed Lab, Politecnico di Torino, Turin, Italy; 3 Department of Neuroscience, Rehabilitation, Ophthalmology, Genetics, Maternal and Child Health (DINOGMI), University of Genova, Campus of Savona, via Magliotto 2, 17100 Savona, Italy; 4 Integrative Physiology Lab, Dept. of Neuroscience, University of Torino, Torino, c.so Raffaello 30, 10125 Torino, Italy

**Keywords:** Neurophysiology, Neurology

## Abstract

It is well established that muscle percussion may lead to the excitation of muscle fibres. It is still debated, however, whether the excitation arises directly at the percussion site or reflexively, at the end plates. Here we sampled surface electromyograms (EMGs) from multiple locations along human vastus medialis fibres to address this issue. In five healthy subjects, contractions were elicited by percussing the distal fibre endings at different intensities (5–50 N), and the patellar tendon. EMGs were detected with two 32-electrode arrays, positioned longitudinally and transversally to the percussed fibres, to detect the origin and the propagation of action potentials and their spatial distribution across vastus medialis. During muscle percussion, compound action potentials were first observed at the electrode closest to the tapping site with latency smaller than 5 ms, and spatial extension confined to the percussed strip. Conversely, during tendon tap (and voluntary contractions), action potentials were first detected by electrodes closest to end plates and at a greater latency (mean ± s.d., 28.2 ± 1.7 ms, p < 0.001). No evidence of reflex responses to muscle tap was observed. Multi-electrode surface EMGs allowed for the first time to unequivocally and quantitatively describe the non-reflex nature of the response evoked by a muscle tap.

## Introduction

The first observations of muscle contraction induced by muscle tap date back to the XIX century. Its complex features have been repeatedly investigated and discussed over the years but are still debated^1–4^. The persisting uncertainty about the actual nature of this phenomenon limits its possible use as bedside neurological test^4–6^.

The present study will not deal with the electrically silent, long-lasting (1–10 s) contraction, also called myoedema or mounding^1,2,7^ that develops upon vigorous percussion of the muscle, particularly in certain pathological conditions^8–11^. This investigation will focus on electrically active, short-lasting (<1 s) contractions, that can be easily elicited in healthy subjects with gentle muscle taps, as reported for different muscles such as vastus medialis (VM)^3,12^, gastrocnemius^2^ and tibialis anterior^4^ in humans and in animal models^2,13^. In particular the origin of the depolarization of muscle fibres in response to a muscle tap have long been debated and no consensus has yet been achieved regarding whether the contraction is *idiomuscular*^1^ or reflex in nature (or both), i.e., whether muscle fibres are excited directly at the percussion site, or indirectly by activation of a reflex arch following stimulation of sensory afferents such as muscle spindles (or both). Convincing evidence of the existence of an idiomuscular component in the tap-evoked contraction was provided by both Meadows^3^ and Brody and Rozear^2^, who showed that the EMG response was not abolished following the interruption of the reflex arc by (i) proximal anaesthetic^3^ or ischemic blockade of the motor nerve in healthy subjects^2,3^, (ii) spinal anaesthesia, in patients undergoing surgical interventions^2^ or (iii) curarization in rabbits^2^, thus supporting the concept that muscle fibres could be directly excited at the percussion site. These authors however did not exclude the occurrence of a reflex response, and in fact a late EMG component of reflex origin was reported to follow the idiomuscular by 36–40 ms after percussing the gastrocnemius muscle^2^. The basis for tap-induced reflex contractions comes from the observation that the muscle taps may activate muscle spindle receptors^14^ and several studies provided evidence supporting the reflex origin of the tap-evoked contraction. Smith *et al*.^15^, showed that 1-mm indentations in masticatory muscles elicited short-latency homonymous and heteronymous EMG activations, compatible with the stretch reflex pathway. Hong *et al*.^13^ observed in the exposed biceps femoris muscle of the rabbit that a twitch contraction was consistently elicited in response to pinch or percussion (by a solenoid-driven blunt metal probe of 0.5 mm of diameter) of the most pressure sensitive point (“trigger spot”), but disappeared after transection of the innervating peripheral nerve^13^.

These conflicting views have been reproposed in two recent studies. The localized contraction elicited by percussing the human VM was detected at a latency (TAP to EMG peak) of about 30 ms by multi-electrode surface EMG^12^ and considered of reflex origin, actually the first evidence of regionalized stretch reflex in humans, an idea originally proposed by Windhorst *et al*.^16^. Conversely, the tap-induced contraction of the tibialis anterior muscle was shown to be preserved during anaesthesia and neuromuscular blockade in neurologically healthy patients^4^, thus again supporting its non-reflex nature, although the occurrence of a late EMG activation, was also reported, in non-paralyzed subjects.

The above cited studies provide evidence for the existence of both idiomuscular and reflex responses to muscle tap but also show a lack of consensus, possibly due to the lack of methodological means to unequivocally detect and characterize the underlying physiological mechanisms. In fact, the two components are too close in time to be discriminated on the basis of force and kinematic recordings and their electrical manifestations cannot be easily discriminated based on single channel EMG recordings.

**Figure 1:**
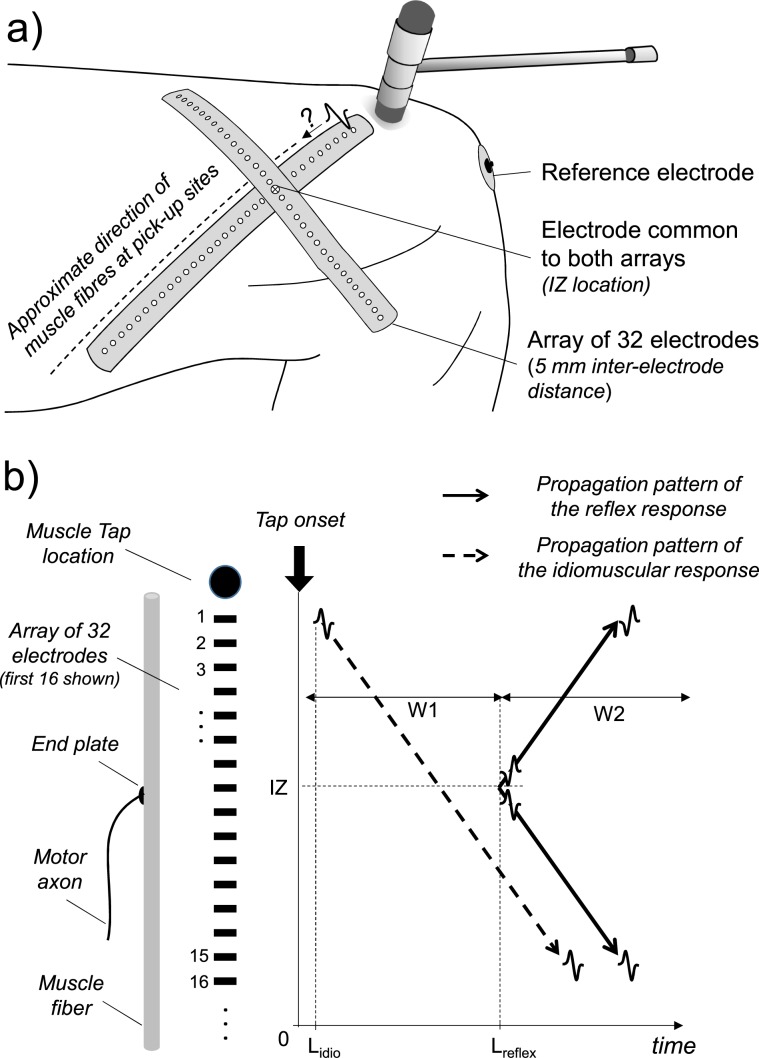
Experimental setup and possible EMG propagation patterns. (**a**) Schematic representation of the experimental setup. Medial view of the human left thigh. Two linear arrays of 32 electrodes (5 mm inter-electrode distance) were positioned longitudinally and transversally with respect to the distal fibres of the vastus medialis (VM) muscle. Muscle taps were delivered at the distal end of the longitudinal electrode array. (**b**) Generation and propagation patterns of EMG potentials associated with the direct idiomuscular (*dashed arrow*) and reflex (*solid arrows*) activation of VM fibres, as detected by the longitudinal array of electrodes. The latencies L (L_idio_ and L_reflex_) between the tap onset and the first detected EMG potentials are shown on the time axis. W1 and W2 are the time windows, within which the amplitudes of the idiomuscular and reflex responses were evaluated, respectively (see methods).

However, the issue can be effectively addressed by detecting surface EMG with multiple electrodes positioned along the whole fibres’ length. This approach can map the origin and propagation of action potentials^17^, as schematically illustrated in Fig. 1. We considered that if the percussion is delivered at one end of the muscle, far apart from the innervation zone, the discrimination between idiomuscular and reflex muscle activation should be straightforward, i.e., originating at the percussed end and propagating to the other end of the muscle in the case of idiomuscular activation (unidirectional propagation pattern, dashed arrow in Fig. 1b) and originating at the innervation zone and propagating toward both ends in case of reflex activation (bidirectional propagation pattern, solid arrows in Fig. 1b). From the hypothetical scheme of Fig. 1b it is also evident that the latency of the response detected from a single electrode pair would not be, per se, sufficient to discriminate between the idiomuscular and reflex response, as both responses may appear with similar latency depending on the detection location. Therefore, understanding the nature of active contractions induced by muscle tap requires the identification of the generation site of the potential, as well as the description of its propagation pattern.

The aim of this study was to assess the origin of tap-induced contractions. To this end, we sampled EMGs from multiple locations along VM fibres. We chose VM because in this muscle tap-evoked contractions can be easily obtained^3,12^, and because its fibres are parallel to the skin surface, thus allowing to sample the evoked potential along the entire fibre length with an array of electrodes. Percussions were delivered to the distal end of the muscle and the EMG response pattern was compared to the reflex EMG response to percussion of the patellar tendon and to the EMG pattern generated by voluntary contractions. A longitudinal array of electrodes was used to detect the origin and pattern of propagation along the relevant muscle fascicle and a transverse array was used to visualize the width of the contracting fascicle (Fig. 1a). In addition, a range of percussion forces were tested to determine whether the magnitude of the EMG response was influenced by the intensity of the stimulus.

## Methods

Five male volunteers (age: 41 ± 5 years, height: 179 ± 4 cm, weight: 72 ± 5 kg) were recruited at the Department of Neuroscience – University of Turin (Turin) for study purposes. No subject had any relevant ongoing neurological or other diseases that may alter muscle activity. The study was conducted in accordance with the Declaration of Helsinki and with the approval of the Ethics Committee of the University of Torino (Approval number: 158423). The informed consent was obtained from all participants prior to the beginning of the study.

### Procedure

The experiment was divided into three sessions executed in the following order: voluntary knee extension contraction, tapping of the patellar tendon (tendon tap, TT), taps of different forces applied to the VM (muscle tap, MT). During all sessions, participants were seated on a chair with backrest and held the knee flexed at 90°. As indicated below, the voluntary contraction was used to identify and localize VM innervation zones. The tendon tap was applied to induce a stretch reflex response of the VM muscle. The sequence of muscle taps was delivered at roughly regular intervals of 2 s, with increasing force from 5 to 50 N and then decreasing again to 5 N in steps of about 5 N. A total of 23–35 muscle taps were delivered to each subject. A resting interval of 5 minutes was provided between sessions.

### Mechanical muscle tap

Muscle taps were delivered by means of a small neurological hammer with a rounded rubber head having a diameter of 13 mm (see below). The taps were delivered at the distal border of VM, close to the patella (see Fig. 1a), where the largest twitch responses could be elicited by minimal taps, as assessed by visual inspection. A piezo-resistive film transducer (Flexiforce, Tekscan, A301) was stuck to the hitting head of the hammer in order to record timing and magnitude of the stimulus. The experimenter held the neurological hammer in place on the muscle while exerting a slight pressure, and delivered the taps by hitting the neurological hammer with a second handheld hammer (weight: 150 g). This technique allowed to 1) considerably reduce stimulus artefacts in EMG recordings and 2) maintain precisely constant the position of the stimulation zone over the muscle, both within the same session, as the neurological hammer was kept in place for its whole duration, and over different sessions, as the neurological hammer could be carefully repositioned over the desired spot, that had been previously marked on the skin.

Given the dependency of the force response of the transducer on the working conditions^18^ its calibration was performed *in situ*, i.e., by loading the neurological hammer with a known axial force (range: 0–50 N).

The tap force was recorded with a system for the acquisition of biomechanical signals (DueBIO, OT Bioelettronica, Torino Italy) and synchronized with the EMG recordings (MEACS Software, LISiN Politecnico di Torino, Torino, Italy). Real-time visual feedback of tapping force was provided to help the experimenter control the hitting strength.

### EMG recordings

The innervation zone and fibre’s direction of the distal VM were identified with a dry linear array of 32 electrodes (5 mm inter-electrode distance) during gentle, isometric contractions at 90° of knee flexion. The position of the innervation zone was defined as the location where phase opposition occurred between consecutive, single-differential EMGs with clear propagation of action potentials toward both extreme electrodes^17^. After identification of the innervation zone, two adhesive linear arrays of 32 electrodes each (20 mm^2^ surface, 5-mm inter-electrode distance) were positioned over the VM (Fig. 1), both secured to the skin with a bi-adhesive foam. One array was placed along the fibres’ direction, with the most distal electrode located just medially to the percussion site. The other array was positioned in a transverse direction, with the 17^th^ electrode located on top of the electrode of the array aligned parallel to VM distal fibres located most closely to the innervation zone (Fig. 1a).

EMG signals were detected in monopolar configuration using a wireless and modular high-density EMG amplifier^19^. This device is composed of two modules, each detecting, conditioning and transmitting 32 EMG signals (gain: 192 V/V; sampling frequency: 2048 Hz; nbits of the AD converter: 16). The two modules transmit the detected sets of signals through a Wi-Fi link to a personal computer for real-time visualization and storage, the synchronization delay between the two modules is smaller than 0.5 ms^19^.

### Data analysis

**Figure 2:**
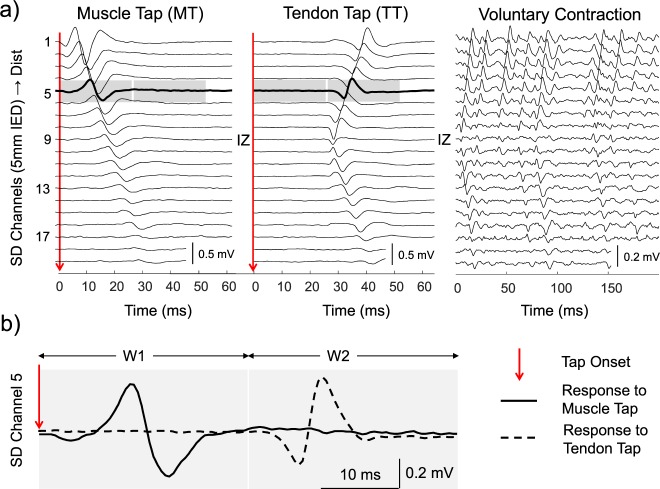
Patterns of propagation of the EMG signal. (**a**) Propagation patterns of experimental, single-differential (SD) EMG responses detected with the longitudinal electrode array in the three experimental conditions: muscle tap (MT), tendon tap (TT), and voluntary contraction. The tap onset is indicated by a *red arrow* in the two panels showing the responses to percussions (MT and TT). IZ indicates the innervation zone position as identified in voluntary and tendon tap contractions. (**b**) EMG potentials induced by muscle tap (*solid line*) and by tendon tap (*dashed line*). The two grey-shaded boxes around EMG responses picked up by electrode 5 indicate the two time windows (W1 and W2), in which the amplitudes of the idiomuscular (W1) and reflex (W2) responses were evaluated.

Monopolar EMGs were band-pass filtered with a second-order Butterworth filter (10–400 Hz cutoff frequencies). Single differential EMGs were then calculated by differentiating monopolar EMGs recorded by consecutive electrodes of array positioned parallel to VM fibres. In this study we considered the possibility that action potentials induced by a mechanical tap could be first detected by the electrode closest to the tap location rather than at the innervation zone (Fig. 1b). In this case, the action potentials would propagate in a single direction, from the distal to the proximal ends of the array aligned to VM fibres (Fig. 1b). Otherwise, action potentials would originate at the innervation zone and propagate bi-directionally toward the fibre ends (Figs. 1b and 2a). These possibilities were systematically investigated by assessing origin, propagation directions, latency and amplitude values of the induced EMG potentials.

To quantify differences between propagation directions, latencies and amplitudes in response to tendon and muscle taps (TTs and MTs, respectively), the following procedures were adopted.

#### Propagation directions

We measured the time delay between consecutive single differential signals detected along muscle fibres^20^. The sign of such delay depends on the propagation direction; hence, if the propagation is unidirectional all delays are expected to have the same sign while if the propagation is bi-directional positive and negative delays can be obtained depending on where in the array the potential is first detected. The delay pattern of muscle taps was compared to that obtained from voluntary contractions, where a bi-directional propagation from the innervation zone to the distal and proximal tendons is expected to occur. Only voluntary contractions were considered for the estimation of delays between consecutive channels because of the relatively low number of reflex responses elicited via tendon tapping across subjects. As for the muscle tap conditions, for which action potentials were successfully elicited for each tap and subject, propagating potentials could be well observed in the surface EMGs during voluntary contractions (Fig. 2a).

#### Latencies

For each tap, we visually identified the electrode where the EMG potential appeared first, and for this electrode we measured the latency between the tap and the EMG onset (Fig. 1b).

#### Amplitudes

The amplitude of the responses evoked by muscle tap and its dependence on the tap peak force were assessed with the root mean square amplitude (RMS) of a single EMG channel. The selected channel, was the one located midway between the innervation zone and the medio-distal end of the array (e.g. channel 5 for the representative subject in Fig. 2). RMS values were computed in two windows (W1 and W2 in Fig. 1), which were defined as the time intervals where the evoked potential was expected to appear if induced at the muscle percussion site (W1) or at the innervation zone (W2). According to this definition, W1 and W2 were two consecutive windows, with W1 stating at the tap onset. The duration of both windows was the latency of the tendon tap responses detected in each subject (Fig. 1b).

The RMS amplitudes of the tap responses were compared with the RMS values of the background noise estimated during the quiescent periods before the tap responses. Specifically, the average + 3std of the RMS values were computed over 20-ms epoch prior to each tap onset. This analysis was performed for all the tap responses in order to (i) describe how the amplitude of the evoked responses changed with the tap force and (ii) determine whether high-force muscle taps were able to induce both a local and a reflex response.

In order to determine the spatial extension of the muscle tap responses, we computed the distribution of RMS values for EMG responses detected by the electrode array positioned transversally to VM fibres. For each tap force, the set of 32 RMS values (20 ms epochs starting at EMG onset defined at the innervation zone channel) was fitted with a Gaussian distribution as described in Gallina and Vieira^21^. The mean and the standard deviation of the optimal Gaussian curve were used to describe the mean location and the transverse dimension of the VM region excited by muscle tapping.

### Statistical analysis

Non-parametric statistics was applied to compare the location and the latency of first action potential appearance in response to muscle tap and tendon tap. Within-subject comparisons between the latency values obtained from muscle tap and tendon tap were assessed with the Mann-Whitney test. For both muscle tap and voluntary contractions, delays between single differential signals detected proximally and distally to the innervation zone were grouped across subjects. Differences in delays between muscle tap and voluntary contractions were evaluated with the Mann-Whitney test. The association between tap force and the amplitude of the evoked potentials in W1 and W2 during muscle tap was quantified with the Pearson correlation coefficient (r).

## Results

### Origin and propagation of action potentials

**Figure 3:**
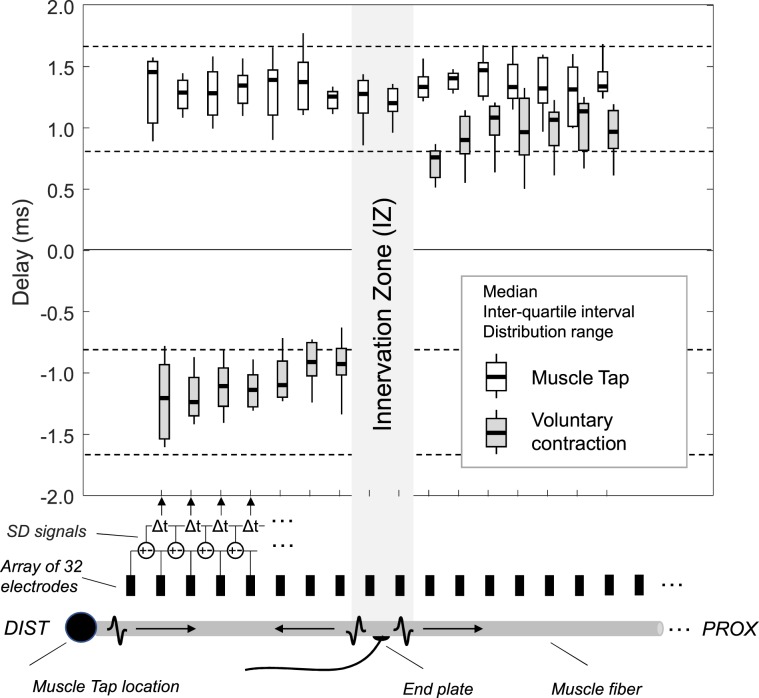
Delays between consecutive single differential EMG signals computed along the fibre direction. Boxplots represent the delay distribution obtained from voluntary contractions (grey boxes) and from muscle taps (MT, white boxes) for all subjects. The sign of the delay depends on the propagation direction. Delays with constant sign along the entire array indicate unidirectional propagation, while a sign change denotes bi-directional propagation. Dashed lines indicate the range of physiological delays associated with a conduction velocity between 3 and 6 m/s. An attenuation of action potentials along the fibres could be observed for all subjects (e.g. Fig. 2a) possibly because of misalignment between fibres and electrodes^24^ and difference in propagation velocity between excited fibres^39^. Therefore the delay values are represented for the maximal number of consecutive channels (n = 16) for which we were able to successfully track the propagation of action potentials.

Potentials induced by muscle tap and by tendon tap originated in different regions of VM. All potentials evoked by muscle-tap (MT potentials) were first detected by the most distal electrode (electrode 1) of the longitudinal electrode array, while all potentials induced by tendon tap (TT potentials) originated at the muscle innervation zone, identified in voluntary contractions (Table 1). Figure 2a depicts a representative propagation pattern of action potentials during voluntary contractions and during both muscle tap and tendon tap. Action potentials observed during voluntary contractions and tendon tap propagate bi-directionally from the innervation zone to the tendon regions, while the propagation of MT potentials was mono-directional, from the electrode close to the percussion site to the proximal electrode. Notably, the propagation patterns of MT and TT potentials (Fig. 2) reproduce the patterns sketched in Fig. 1b for the idiomuscular and reflex responses, respectively. Difference in the propagation direction of EMG signals between muscle tap and voluntary contraction conditions was quantitatively assessed through the analysis of the delay between consecutive single differential signals. Box plots in Fig. 3 show that while absolute delays were roughly similar for both conditions (p < 0.05 distally and p > 0.05 proximally to the innervation zone), signals detected distally from the innervation zone provided negative values for voluntary contractions and positive values for muscle tap.

**Table 1:** Origin of the compound action potential.

Subject N	EMG channel where the potential originates		
	Muscle Tap	Tendon Tap	Voluntary
1	1	6	6
2	1	8	8
3	1	9	9
4	1	9	9
5	1	9	9

### Latency of the evoked potentials

**Figure 4:**
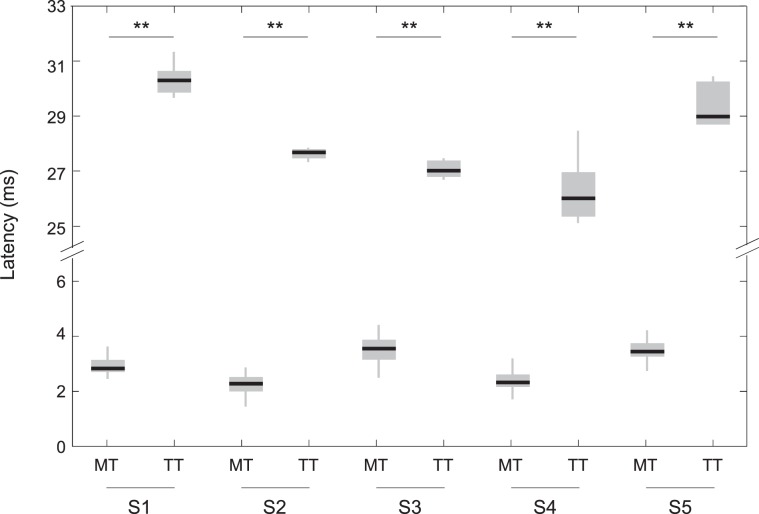
Latencies between the tap and EMG onsets for muscle tap (MT) and tendon tap (TT). Data is shown for each individual subject. Note the broken ordinate. (**p < 0.001, Mann-Withney test).

The latency between the tap onset and the first observed potential differed for the two tapping conditions. The average latency across subjects and trials was 3.6 ± 1.7 ms for muscle tap and 28.2 ± 1.7 ms for tendon tap. The analysis on individual participants showed statistically significant differences in latency between muscle tap and tendon tap (p < 0.001 for all subjects, Fig. 4). As can be inferred from the representative recordings of Fig. 2, the latency of the responses depends on the channel number, i.e., its actual position along the muscle. For instance, the latency between the tap onset and the occurrence of the evoked potential at the innervation zone channel number 9 was 18.6 ± 1.9 ms for muscle tap and 28.2 ± 1.7 ms for tendon tap.

### Effect of the tap force on the amplitude of muscle tap potentials

**Figure 5:**
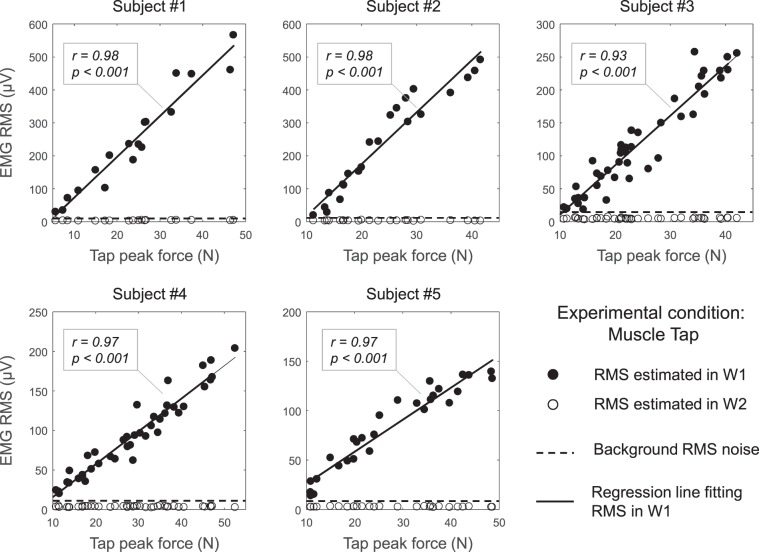
Effect of the muscle-tap force on the induced EMG amplitude for each participant. RMS values were computed for two windows (W1 and W2, see Fig. 2) defined as the time intervals where the induced potential was expected to appear if induced at the muscle tap location (W1) or through a reflex (at the IZ channel – W2). For each graph, the *dashed horizontal line* indicates the background noise level, while the *solid line* is the regression line fitting RMS estimates in W1. Pearson correlation coefficients (r) are reported within each scatter plot.

The lowest tap force able to induce an EMG response larger than the background noise level ranged from 5.6 N (Subject 1) to 11.3 N (Subject 2). As shown in Fig. 5, the EMG amplitude estimated in W1 (representing the amplitude of the idiomuscular component of the response) increased linearly with the muscle tap force (Pearson r > 0.93; p <0.001, for all the subjects, Fig. 5). By contrast, the amplitude of the EMG signal in W2 (representing the amplitude of the potential reflex response), did not exceed the background noise level (dashed, horizontal line in Fig. 5) regardless of the tap force applied to the muscle.

### Location and transversal dimension of the region excited by muscle tapping

**Figure 6:**
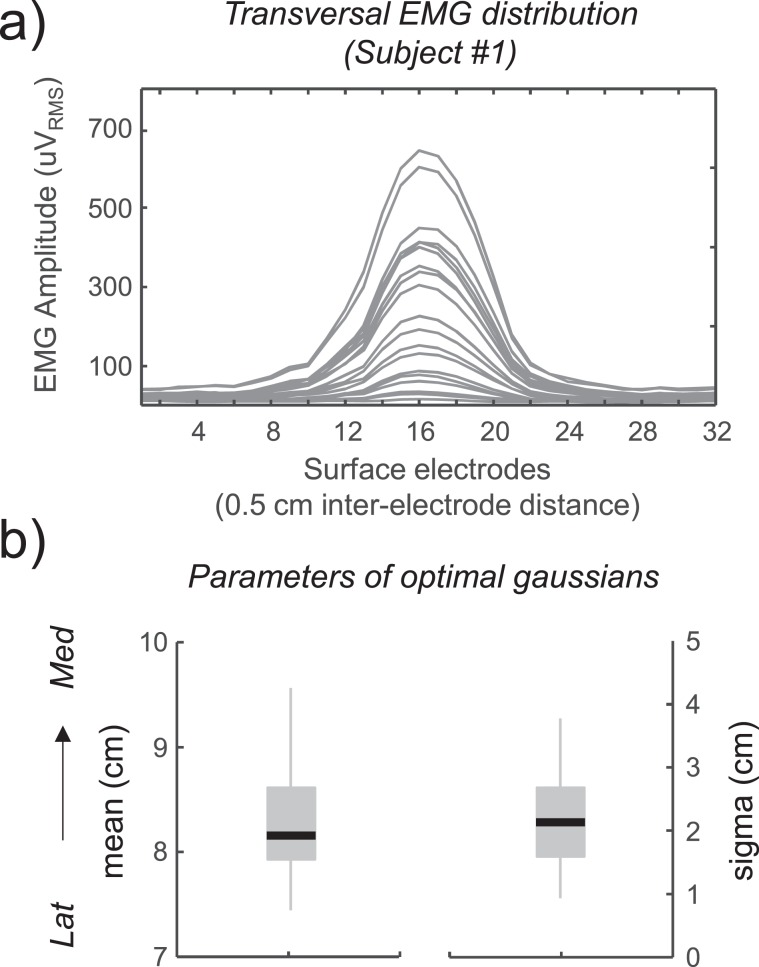
Transversal distribution of the EMG signal in response to muscle tap. (**a**) Amplitude distributions of the EMG responses induced by muscle tap and detected by the transversal electrode array for one representative subject. Different traces represent the amplitude distributions for different muscle tap forces. The abscissa refers to the numbers of electrodes and therefore spans 16 cm. (**b**) Average values of means and standard deviations of the Gaussian distributions fitting the transversal amplitude profiles for all subjects and tap forces. The ordinate refers to the medio-lateral position along the transversal electrode array (med = medial; lat = lateral).

The EMG response distribution derived from the electrode array positioned transversally to VM fibres showed a similar profile across subjects. Typically, EMG responses with the largest amplitudes were detected at the centre of the transversal array (electrode 17) and decayed progressively towards the most external electrodes of the array. This behaviour is exemplified by the EMG distributions of an individual subject shown in Fig. 6a. Figure 6b shows the average values, across subjects and tap forces, of the mean and standard deviation of the Gaussian distributions fitting the transversal amplitude profiles. The whisker plots in Fig. 6b show that EMG distributions were centred in the middle of the transversal array (mean [range]: 8.3 [7.5–9.5] cm) with an average dispersion of 2 cm [1.0–3.8] cm.

## Discussion

The present study assessed the pattern of generation and propagation of action potentials in vastus medialis (VM) muscle in response to muscle taps as compared to tendon taps. The results demonstrate that when the tap is delivered to the distal end of VM fibres, the induced potential arises immediately at the distal percussion site and then propagates proximally, towards the other end of the muscle fascicle. The propagation pattern is thus completely different then during the reflex muscle contraction, that is generated at the innervation zone at a much longer latency and propagates bidirectionally (Fig. 4 and Table 1).

The EMG propagation patterns evoked by muscle taps demonstrate their “idiomuscular”^1^ rather than a reflex origin. This qualitative observation is further supported by the quantitative assessment of the propagation delay of EMG signals across consecutive electrodes (Fig. 3). Figure 3 also seems to indicate a slightly higher conduction velocity for voluntary contractions, compared to muscle tap (MT). This could be due to changes in the muscle membrane properties, the different alignment between fibres and the electrode array or both. Specifically, increase in conduction velocity is known to take place in muscle fibres upon repetitive firing (as with voluntary contractions) as compared to single excitations (as with muscle taps)^22,23^. Regarding the misalignment issue^24^, it should be noted that while during voluntary contractions the vastus fibres were slightly loaded, during muscle tap the fibres were at rest. However, delay values were well within the physiological range for skeletal muscles (Fig. 3). Most importantly, action potential propagation was unidirectional during muscle tap; this is not compatible with the generation of action potentials at the VM fibres end plates during muscle tap.

The unidirectional propagation of action potentials during muscle tap supports and integrates the evidence collected in old and recent studies in which the tap-evoked muscle excitation and contraction were shown to persist after ischemic or anaesthetic blockade of the motor innervation^2,3^ and even after muscle curarization^2,4^. A short comment is here required to discuss why in the same experimental conditions this contraction was recently interpreted as the demonstration of a localized reflex response^12,25^. In their study Gallina and colleagues^12^ systematically positioned the electrode matrix proximal to the innervation zone, i.e., over an area in which the propagation patterns of reflex and idiomuscular responses have the same direction and cannot be used to discriminate between the two. In fact, as depicted in Fig. 1 and demonstrated quantitatively in Figs. 2 and 3, the two responses present opposite directions of propagation only in the muscle portion between the percussion site and the innervation zone. It seems that the authors based their assumption about the reflex nature of the EMG responses on the observation that their latencies (about 30 ms) were compatible with a stretch reflex^12^. However, we here observed that the latency of the idiomuscular EMG response is approximately 18 ms when recorded at the innervation zone. This value may plausibly increase to 30 ms if recorded a few cm more proximally or if the EMG onset is measured at the peak rather than at the foot of the EMG wave^12^.

The issue of assessing the latency of the reflex EMG response to a stimulus is not trivial. While it is quite obvious that the conduction time along the neural afferent and efferent pathways constitutes a relevant portion of the delay (proportional to the distance between the stimulated area and the spinal cord), the conduction time of muscle fibres is often neglected. However, this latter component introduces an additional delay of 2.5–3.3 ms per centimetre of muscle fibre (hypothesizing a conduction velocity of 3–4 m/s), which sums up to a relevant delay if the distance between recording electrodes and innervation zone is large. Depending on the muscle length and the experimental setting (percussion site, innervation zone, recording site), the propagation scheme of Fig. 1b may change in that the dashed line intersects the continuous line. In other words, at certain recording locations, the muscle tap response may exhibit larger latency than the tendon tap response. Proper assessment of muscle conduction time requires knowledge of the location of the innervation zone, of the orientation and of the conduction velocity of muscle fibres, which in turn may be affected by the non-cylindrical shape of muscle fibres^26^. In this respect, surface EMG recordings with array- or matrix-electrodes provide invaluable help for collecting all these data at once. With our multi-electrode approach, we fully characterised the response to VM percussion. With the longitudinal array, we demonstrate that fibres are excited at the site of percussion (Figs. 3, 4 and Table 1) whereas with the transverse array we show that percussion excites only fibres located beneath the stimulation site (Fig. 6).

It should be noted that in all the 180 taps delivered to VM, action potentials were first detected by the electrode closest to the tapping location rather than at the end plate. These results indicate that the nature of tap responses cannot be ascertained from EMGs detected from a single location, without knowing its position with respect to the muscle innervation zone. The relevance of this consideration is unlikely to change should additional subjects be tested. In fact, detecting possible exceptions to the observed patterns of excitation would not disprove the results of this study.

We were interested in understanding whether, besides the idiomuscular EMG response, a reflex response could also be evoked by the tap stimulus, as reported in other studies^2,3^. To this end, a large range of stimulus intensities was tested. While the magnitude of the idiomuscular component exhibited a linear dependence on the stimulus intensity (Fig. 5), no additional EMG response beyond this earlier response was ever noticed, even at the highest stimulus intensities. In particular, no evidence of increased EMG activity was detected in the time window W2, which should have contained the reflex response (open circles in Fig. 5), as individually established by observing the latency of the response to the tendon tap (as explained in Fig. 2). This is partly in contrast with previous studies in which the occurrence of a reflex component was reported^2,3,13^. However, the likelihood of observing a reflex response may depend on several factors such as: (1) the specific anatomy (origin, insertion and arrangement of muscle fibres) of the muscle investigated; (2) the amount of muscle mass affected by the stimulus, which may also depend on the size of the hammer head; (3) the length of the muscle (angle of joint angle(s) spanned), which co-determines the relative arrangement of muscle fibres and pre-stretch of muscle spindles; (4) the degree to which the percussion translates into stretch of muscle fibres and activation of in-dwelling muscle spindles; (5) static background contraction and hence fusimotor activation of muscle spindles, which usually facilitate reflex responses; and (6) the fraction this mass represents of the whole muscle or muscle group. In particular, the strip of VM stimulated by the muscle percussion is very small compared to the muscle mass stimulated by a tap delivered to the patellar tendon. This proportion is likely to increase when tapping the gastrocnemius muscle as in Brody and Rozear^2^ and the Achille’s tendon. These factors may possibly explain why a reflex EMG response to muscle tap was reported in some studies^2,3,13^ but not observed here.

Meadows^3^ also observed that the stimulated muscle fibres sometimes produced a prolonged series of action potentials (burst activation). No evidence of such repetitive firing was here detected by surface EMG, possibly because of its mostly occasional and asynchronous nature. On the contrary, the surface EMG response always exhibited a clean and stereotyped pattern, indicative of single action potentials, synchronously excited in a population of muscle fibres.

To our knowledge the mechanisms underlying the tap-evoked excitation of muscle fibres have not been investigated, nor can they be inferred from the results of the present study. We may just observe that action potentials in muscle fibres are in fact immediately generated upon percussion (see Fig. 2). It may be speculated that the abrupt sarcolemmal deformation produced by the percussion opens mechano-sensitive ion channels^27^ and activates depolarizing currents that excite the affected muscle fibres, similarly to what has been described for nerve fibres^28,29^. In support of this hypothesis is the abundance of mechano-sensitive channels on the sarcolemma, belonging to the *transient receptor potential* (TRP) and to the *Piezo* families^30,31^. They are considered to be sensitive to the force transmitted by the lipid bilayer of the cell membrane^32^ and, in particular, to shear stress and stretch of the membrane^33^. In the skeletal muscle, these non-selective cation channels are considered to have a role in different processes such as muscle development and contrast to muscle fatigue. In addition, due to their contribution to calcium inflow they have been implicated in the alteration of the contractile machinery in muscle dystrophies^30,31,34^. In vascular smooth muscle, stretch-activated mechano-sensitive cation channels are considered to mediate the membrane depolarization that precedes the contraction, in the myogenic response (the vessel constrictory response to increased transmural pressure)^35^. To our knowledge, whether mechano-sensitive channels may mediate a depolarization, and possibly the excitation, of mechanically-stimulated skeleletal muscle fibres has not been investigated, but it seems a plausible explanation that the massive and synchronous opening of mechanosensitive cation channels produced by the sharp percussive stimulus may cause a local depolarization of sufficient magnitude to excite the muscle fibres and, thus, lead to the idiomuscular contraction.

The propensity of muscle fibres to depolarize and contract upon mechanical perturbation may have implications in the field of neurology and rehabilitation. The adoption of muscle tap as a bedside neurological test is implemented and/or suggested by several studies to support and speed-up the diagnostic process^4–6,36^ while at the same time acknowledging that a better understanding of the mechanism underlying the contractile response would further improve its usefulness. In the field of musculoskeletal disorders contractions evoked by mechanical stimulation of the muscle imparted by the practitioner are considered relevant signs for the diagnosis of musculoskeletal disorders^37,38^. For instance, the occurrence of the *twitch response* following a *snapping manoeuvre* (stimulation of the muscle with the fingertips of the index and the thumb finger producing force transversally to the fibres) or muscle tap^13,37^ is among the signs required for the identification of *trigger points*^38^ and is generally believed to be of reflex origin^13^. Based on the present results and on the similarity between snapping and tapping stimuli, we believe this hypothesis should be carefully tested by means of multi-electrode surface EMG recording.

In conclusion, we demonstrated that muscle tap elicits an immediate excitation of muscle fibres at the percussion site. The population of excited fibres linearly increases with the stimulus intensity but remains confined to the muscle band affected by the mechanical stimulus. On the contrary, reflex EMG activation was not observed, not even at very large stimulation intensities. The methodological approach adopted, based on electrode-array surface EMG, allows to compare origin and propagation pattern of EMG signals during voluntary, reflex and tap-evoked idiomuscular contractions and paves the way to investigate these issues in other muscles and under different conditions.

## Data Availability

The datasets generated during and/or analysed during the current study are available from the corresponding author on reasonable request.
